# Understanding Rate and Capacity Limitations in Li–S Batteries Based on Solid-State Sulfur Conversion in Confinement

**DOI:** 10.1021/acsami.4c13183

**Published:** 2024-11-29

**Authors:** Ayca Senol Gungor, Jean-Marc von Mentlen, Jean G. A. Ruthes, Francisco J. García-Soriano, Sara Drvarič Talian, Volker Presser, Lionel Porcar, Alen Vizintin, Vanessa Wood, Christian Prehal

**Affiliations:** † Department of Information Technology and Electrical Engineering, ETH Zürich, Gloriastrasse 35, 8092 Zürich, Switzerland; ‡ INM—Leibniz Institute for New Materials, Campus D2 2, 66123 Saarbrücken, Germany; § Department of Materials Chemistry, National Institute of Chemistry, Hajdrihova 19, 1000 Ljubljana, Slovenia; ∥ Department of Materials Science and Engineering, Saarland University, Campus D2 2, 66123 Saarbrücken, Germany; ⊥ Saarene–Saarland Center for Energy Materials and Sustainability, Campus C4 2, 66123 Saarbrücken, Germany; # Institut Laue−Langevin, 71 Avenue des Martyrs, 38042 Grenoble, France; ¶ Department of Chemistry and Physics of Materials, Paris-Lodron University of Salzburg, Jakob-Haringer-Straße 2a, 5020 Salzburg, Austria

**Keywords:** Lithium−sulfur batteries, nanoporous carbons, operando scattering, impedance spectroscopy, solid-state sulfur conversion, electrochemical performance

## Abstract

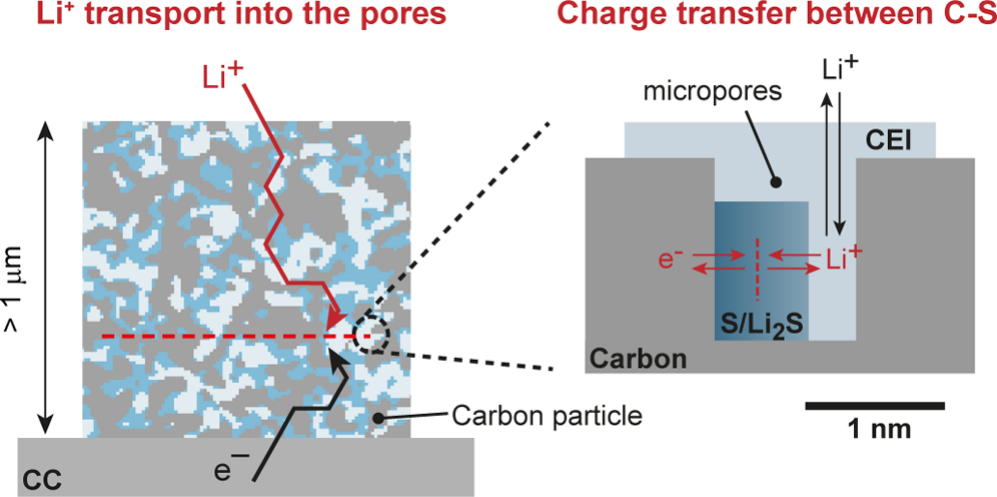

Li–S batteries with an improved cycle life of over 1000 cycles have been achieved using cathodes of sulfur-infiltrated nanoporous carbon with carbonate-based electrolytes. In these cells, a protective cathode–electrolyte interphase (CEI) is formed, leading to solid-state conversion of S to Li_2_S in the nanopores. This prevents the dissolution of polysulfides and slows capacity fade. However, there is currently little understanding of what limits the capacity and rate performance of these Li–S batteries. Here, we aim to deepen our understanding of the capacity and rate limitation using a variety of structure-sensitive and electrochemical techniques, such as *operando* small-angle neutron scattering (SANS), *operando* X-ray diffraction (XRD), electrochemical impedance spectroscopy, and galvanostatic charge/discharge. *Operando* SANS and XRD data give direct evidence of CEI formation and solid-state sulfur conversion occurring inside the nanopores. Electrochemical measurements using two nanoporous carbons with different pore sizes suggest that charge transfer at the active material interfaces and the specific CEI/active material structure in the nanopores play the dominant role in defining capacity and rate performance. This work helps define strategies to increase the sulfur loading while maximizing sulfur usage, rate performance, and cycle life.

## Introduction

Lithium–sulfur (Li–S) batteries are promising candidates to outperform current Li-ion batteries in terms of cost, environmental friendliness, and storage capacity.^1,2^ However, they are not yet largely commercialized because of poor cycle life^3−6^ and problems reaching their theoretical capacities.

In standard Li–S batteries with ether-based electrolytes,^7^ the conversion from sulfur (S) to Li_2_S is realized via a solid–liquid–solid process.^8^ During discharge, solid sulfur is reduced to soluble polysulfides, eventually precipitating to solid Li_2_S.^9,10^ Dissolving the polysulfides improves the reaction kinetics^11^ compared to a solid-state conversion but requires high amounts of electrolyte,^12^ which significantly reduces the practical cell capacities and energy densities. In addition, polysulfides cause self-discharge, active material loss, and capacity degradation.^3,13,14^

These issues can, in principle, be circumvented with Li–S batteries using sulfur-infiltrated nanoporous carbon (pores <2 nm) and carbonate-based electrolytes, where conversion from S to Li_2_S happens in the solid state.^15^ The solid-state conversion is enabled by the in situ formation of a cathode–electrolyte interphase (CEI) during the first discharge^16−19^ from a nucleophilic reaction of soluble polysulfides with carbonates and the electrochemical formation of CEI components such as lithium fluoride (LiF).^20−25^ The CEI prevents the further dissolution of S into polysulfides, enabling a direct conversion between solid S and solid Li_2_S in the carbon nanopores.^26^ As polysulfides in the bulk electrolyte are largely avoided, the cycle life compared to Li–S batteries with ether-based electrolytes is improved, and in principle, practical energy densities are increased due to lower electrolyte-to-sulfur (E/S) ratios. However, while the nanoporous carbon supports the formation of the CEI, mitigates polysulfide shuttling,^16^ and offers good electronic conductivity, the limited pore volume leads to low S loadings.^27^ An open challenge is therefore to increase S loading, thereby achieving high capacities while reaching high rates and a long cycle life. To do so, a better understanding of the capacity and rate-limiting processes in these types of Li–S batteries is needed.

Several hypotheses exist about what drives solid-state conversion in Li–S cells using S-infiltrated nanoporous carbons and carbonate electrolytes. Some early works argued that the confinement in pores is the main reason for the solid-state conversion.^28−30^ Smaller pores would only host the small S allotropes like S_2–4_ and no solvent molecules; therefore, they are suppressing polysulfide formation/dissolution.^4,31^ However, later studies indicated that solid-state conversion is also possible in pores >1 nm^16,32^ as long as carbonate electrolytes form a CEI.^17,27,33,34^ Several X-ray photoelectron spectroscopy (XPS) studies have shown the presence of thiocarbonates, alkyl carbonates, lithium carbonate (Li_2_CO_3_), and LiF on the carbon–sulfur cathodes^21,26,35,36^ but no long-chain polysulfides.^5^ X-ray absorption spectroscopy indicated no intermediate polysulfides formed during further cycling with carbonate electrolyte.^27^ These results suggest that the pore structure facilitates the encapsulation of CEI and solid-state conversion.

Overall, the mechanisms of CEI formation, solid-state conversion, and the relation between the structure, transport, and performance are still poorly understood. There is no consensus on what limits capacity and rate performance.

This study aims to gain more insight into where CEI forms and the capacity and rate-limiting processes. We use a combination of techniques: *operando* X-ray diffraction (XRD), *operando* small-angle neutron scattering (SANS), electrochemical impedance spectroscopy (EIS), galvanostatic intermittent titration technique (GITT), and galvanostatic cycling. We systematically vary parameters such as the activated carbon pore size (AC08 with a 0.8 nm pore size and AC12 with a 1.2 nm pore size), the sulfur loading in the C/S cathodes, and the cycling rate.

## Experimental Section

### Materials

Elemental sulfur (powder, 99.98% trace metals basis, Sigma-Aldrich and without any further processing) and nanoporous activated carbons (dried at 200 °C under vacuum overnight), MSP20 (denoted as AC08), and YP80F (denoted as AC12) were mixed manually with different weight ratios in an agar mortar. AC08 and AC12 have mean pore sizes of about 0.8 and 1.2 nm, respectively, provided by Kansai Coke and Chemicals and Kuraray Chemicals Co.

Before melt infiltration, the AC08 and AC12 carbons were mixed with elemental sulfur at different sulfur-to-carbon mass ratios: 2:1, 1:1, and 1:2. The prepared mixture was melt-infiltrated at 155 °C for 7–8 h in a sealed evacuated glass oven (Büchi, Switzerland). The final sulfur mass content was verified by weight and thermogravimetric analysis (TGA). TGA results indicate that the nominal C/S ratios are kept during melt infiltration (Figure S1). For the higher C/S ratios (C/S 1/2), a certain fraction of sulfur is present outside the nanopores. The specific pore volume before and after sulfur infiltration further confirms that sulfur fills primarily the nanopores (Figure S2).

The free-standing film electrodes were prepared by mixing carbon with polytetrafluoroethylene (PTFE, 60 mass % suspensions in water, Sigma-Aldrich) at 9/1 mass ratio with isopropanol (≥99.8%, Sigma-Aldrich). The resulting dough-like material was rolled into a 50–80 μm thick film and dried at 50 °C under vacuum (10 mbar) for 2 h.

After drying, the electrodes were cut (puncher diameter of 13 mm), resulting in a geometrical surface area of 1.32 cm^2^. The sulfur loadings varied from 1.7 to 6.0 mg_S_ cm^–2^, depending on the S/C ratio. As an electrolyte, a solution of 1 M lithium hexafluorophosphate (LiPF_6_) in fluoroethylene carbonate (FEC): dimethyl carbonate (DMC) (by volume 1:4) was used. All solvents were dried with molecular sieves (3 Å, beads, 8–12 mesh, Sigma-Aldrich), and the salt was dried under a vacuum overnight.

### Methods

The gas adsorption measurements were conducted at the INM Saarbrücken. To examine the effect of sulfur infiltration into the carbon structure, nitrogen adsorption analyses at −196 °C were carried out by using a Quadrasorb IQ system (Anton Paar, formerly Quantachrome). Before each measurement, the pristine carbon materials were outgassed for 12 h at 300 °C, while the infiltrated samples were outgassed for the same duration at 50 °C, both under vacuum. A quenched solid density functional theory (QSDFT) approach, part of Quadrasorb IQ software, was applied to determine the cumulative specific surface area and pore size distribution (PSD). Specifically, the QSDFT with slit-pore geometry was used to determine the PSD.^37^ The pore volume was derived from the cumulative PSD data of up to 35 nm. The average pore size is defined as the pore size at which half of the total pore volume is reached.

TGA to determine the sulfur content was performed on an STA 449 F3 Jupiter under an argon atmosphere, with a heating rate of 10 °C min^–1^ up to a maximum temperature of 900 °C.

All custom-built coin-cell-type electrochemical cells were assembled under an inert atmosphere in an argon-filled glovebox. The cells consisted of an aluminum current collector (18 mm in diameter), a free-standing C/S cathode, a glass fiber separator (20 mm in diameter, Whatman GF/A glass microfiber filters), and a metallic lithium anode (18 mm in diameter, 110 μm thick, FMC Lithium corporation). The electrolyte-to-sulfur ratio was kept at 20–40 μL mg_S_^–1^ to ensure that the electrolyte amount does not limit the electrochemical performance.

All electrochemical characterization was performed with a Biologic VMP3 or MPG2 potentiostat/galvanostat. Galvanostatic cycling was done between 3.0 and 0.5 V vs Li/Li^+^ with a rate of C/10 rate (0.167 A/g_S_, 0.3–1 mA/cm^2^ depending on the loading, except for the rate performance tests). The lower potential limit of 0.5 V was chosen to achieve the maximum capacities, in line with a previous study.^4,38^ During rate capability measurements, the first discharge was done at a rate of C/10, and then three charge/discharge cycles were completed at each rate.

EIS measurements were conducted in potentiostatic mode during GITT. For every 100 mA h g_S_^–1^ (every 200 mA h g_S_^–1^ during the first discharge), the cells were rested at the open-circuit voltage (OCV) for 1 h; then the impedance spectrum was measured between 1 MHz and 0.5–1 mHz with a 5 mV perturbation amplitude. Relaxation times at OCV were chosen for 1 h before EIS measurements started. The relaxation voltage vs time indicates sufficient equilibration while maintaining experimental efficiency.

Samples for scanning electron microscopy (SEM) were extracted from the cells, washed with diethylene glycol dimethyl ether (2G, anhydrous, 99.5%, Sigma-Aldrich), and dried under a vacuum. All steps were completed in an Ar-filled glovebox, and the samples were transferred to the SEM instrument inside a vacuum transfer holder. SEM micrographs were taken with a Hitachi SU-8200 at 1 kV acceleration voltage with backscattered and secondary electron detectors.

*Operando* SANS measurements during galvanostatic cycling were conducted at the D-22 SANS beamline at the ILL neutron source (Grenoble, France). The experimental setup was identical to a previously used setup^39^ (Figure S3). A wavelength of 0.5 nm, a beam diameter of 10 mm, and two areal detectors (sample-to-detector distance of 17.6 and 1.4 m) were employed to ensure an overlapping *q*-region.^40^ The custom-built *operando* SANS cell (Figure S4) is identical to the previously used SANS cell.^39^ Aluminum windows, each with a diameter of 12 mm, were employed to maintain a low background and uniform pressure throughout the cell assembly. The cell assembly comprised a copper foil current collector (≥99.9%, Schlenk Metallfolien), a Li metal anode (≥99.9%, Alfa Aesar, 0.75 mm thickness, 16 mm diameter), a glass fiber separator (Whatman GF/A, 21 mm diameter, 260 μm thickness), an AC08/S cathode (13 mm in diameter, 180 μm thick), and an aluminum current collector (≥99.5%, Korf). The assembly was infilled with 200 μL of electrolyte (1 M LiPF_6_ in FEC/DMC_deuterated_, in a volume ratio of 1:4). The neutron beam irradiated all components of the cell, but discernible and reversible structural alterations were only observed in the cathode.

**Figure 1:**
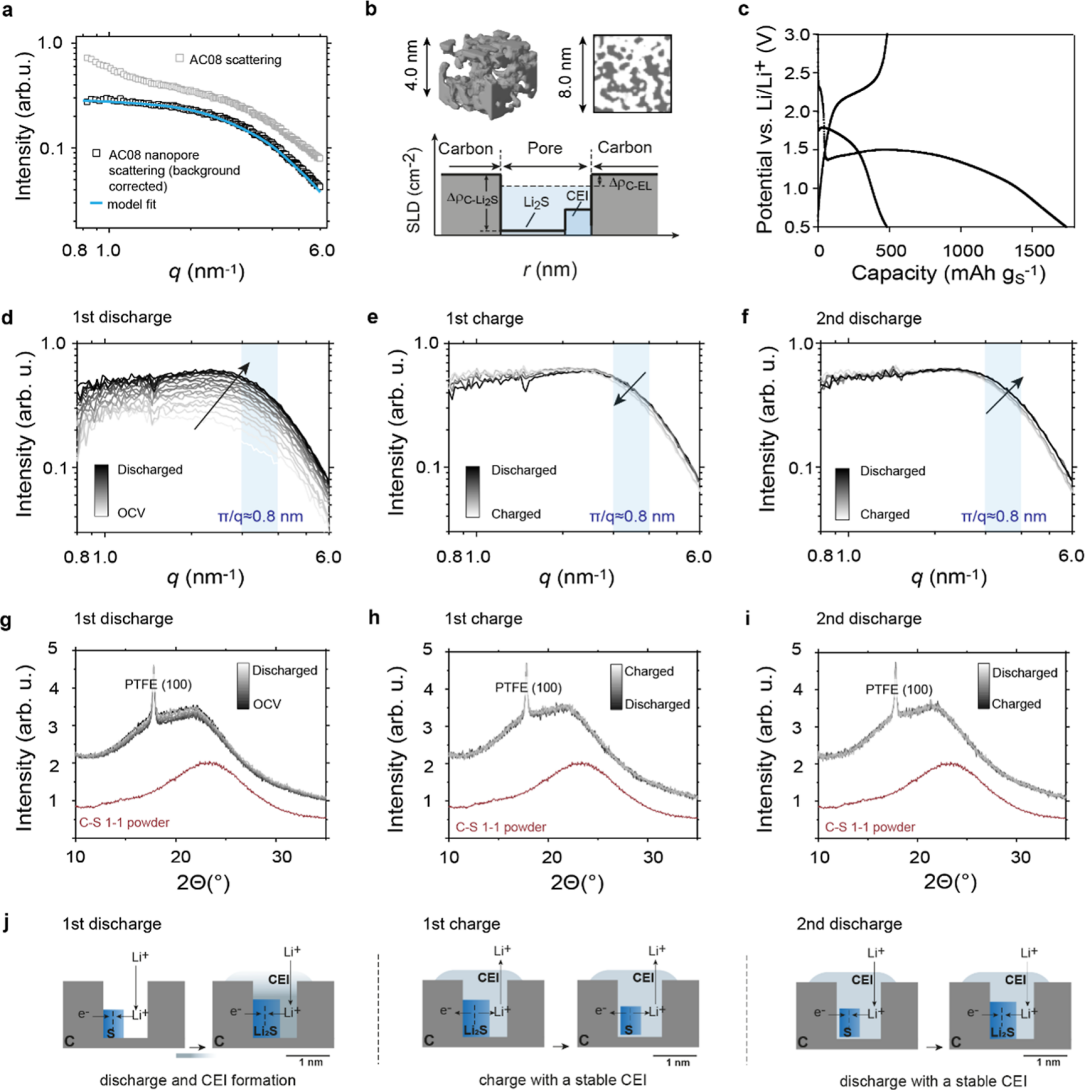
*Operando* SANS and XRD measurements. (a) SAXS intensity of the AC08 carbon (gray) versus momentum transfer *q*, after subtraction of the particle scattering at low *q* and the subtraction of a constant background (black). The blue solid line corresponds to a model fit based on GRFs. The data is taken from ref (41). (b) Top: 3D and 2D visualization of the AC08 nanopore structure based on the GRF model fit in (a).^41^ Bottom: sketch of SLDs of different phases in the C–S composite. Detailed numbers are given in Table S1. (c) Galvanostatic (dis)charge curves of the in situ SANS cell (AC08/S cathode with C/S ratio of 1/1). (d) SANS intensities versus scattering vector length *q* during the first discharge. An increasing SLD difference (as shown in b) and the resulting SANS intensity increase indicate the CEI formation. (e) SANS intensities versus scattering length *q* during the first charge. (f) SANS intensities versus scattering length *q* during the second discharge. *Operando* XRD intensity of an identical cell (AC08/S ratio of 1/1) during the (g) first discharge, (h) first charge, and (i) second discharge. The absence of sharp crystalline S diffraction peaks during any cycling step confirms the solid-state conversion inside the nanopores. The XRD pattern of the pristine C/S powders is given in red. (j) Schematic summary of the processes occurring during the first discharge, first charge, and second discharge. The conversion takes place without causing any changes in nanopore structure and with no crystalline products.

The 2D detector intensity signal was subjected to azimuthal averaging, corrected for sample holder scattering and electronic background, and then normalized using transmission values. The corresponding SANS intensities and electrochemical data are shown in Figure S5 and Figure 1. To determine the low-*q* background mainly originating from the AC08 particle scattering, we fitted a power-law model of the form *I*_mod_(*q*) = BG_0_ + *I*_0_*q*^–α^ to the average of five consecutive SANS curves right after the high-voltage plateau at 2.3 V vs Li/Li^+^ during the first discharge (see Figure S5a).^41^ After the high-voltage plateau, S has at least in parts dissolved into polysulfides, and Li_2_S and CEI have not yet formed. What remains is approximately the scattering from nanopores and any nanostructure inside the nanopores.^39,42^ We subtracted the so-determined power-law term from all SANS intensities (assuming it would remain approximately constant during cycling). In the next step, we subtracted a flat background originating from diffuse/incoherent scattering. To determine the background (BG) for each recorded SANS intensity (Figure S5b), we fitted the SANS intensities between 5 and 6 nm^–1^ with a Porod power law decay of the form *I*_mod_(*q*) = BG + *Pq*^–4^ (Figure S5b). The resulting background-corrected data are given in Figure S5c. The SANS intensities in Figure 1 are averaged over five consecutive SANS intensities to reduce the noise and improve visualization.

The *operando* XRD measurements were conducted with an equivalent custom-made *operando* cell design adjusted for X-rays in transmission mode (Figures S3 and S4). A small hole (2 mm) protected by a Mylar window guaranteed the penetration of the primary X-ray beam and diffracted X-rays. The cells comprised a Li metal anode, electrolyte (1 M LiPF_6_ in FEC/DMC, v/v 1/4), separator, and the S–C composite cathode (with S loading 3.84 mg_S_/cm^–2^ and the corresponding E/S ratio 40 μL mg_S_^–1^). During in situ XRD measurements, a Biologic SP240 potentiostat/galvanostat was used for electrochemical cycling. Ex situ and in situ XRD measurements were carried out on a Rigaku SmartLab 9 kW system, with a rotating Cu anode and 2D solid-state detector (HyPix-3000 SL).

## Results and Discussion

### Operando Small-Angle Neutron Scattering and XRD

To understand structural changes of the nanoporous activated carbon–sulfur during galvanostatic cycling, the AC08-S cathode (C/S ratio of 1:1) was first studied via *operando* SANS and *operando* XRD. The small-angle scattering (SAS) intensity in Figure 1a (data taken from an ex situ small-angle X-ray scattering (SAXS) measurement in ref (42)) shows the scattering contribution of the empty AC08 nanopore structure. The hump at approximately 3–4 nm^–1^ indicates a mean pore size of approximately 0.85 nm (π/3.7 nm^–1^ ≈ 0.85 nm), which fits the mean pore size determined via gas adsorption (0.8 nm)^41^ (Figure S2). Using Gaussian random fields (GRFs), we generate a statistically representative real space nanopore structure (Figure 1b, top) from the SAS model fit in Figure 1a. The scattering length density (SLD) difference between the carbon matrix and pore (Figure 1b, bottom) determines the absolute intensity value of the scattering curve. The SLD values sketched in Figure 1b correspond to the SLD values for the *operando* SANS measurements. If the nanopores are filled with a material of random shape and distribution, the entire SAS curve will retain its shape but shift on the logarithmic intensity axis. If a new structure appears outside the pores with a length scale larger than ≈1.5 nm and smaller than ≈20 nm, additional features at *q* < 2 nm^–1^ would emerge.

The galvanostatic (dis)charge profiles of the *operando* SANS and *operando* XRD measurements are given in Figure 1c and Figure S6, respectively. The first discharge shows an irreversible capacity slightly higher than the theoretical capacity of sulfur, which can be explained by CEI formation.^16^ The shape of the later charging and discharging cycle without a second high-voltage plateau gives evidence for solid-state conversion.^43^

During the first discharge, the SANS intensity increases by about a factor of 4 without a significant change in the shape. This increase indicates solid Li_2_S and CEI components forming in the nanopores, replacing S and electrolyte^44^ (Figure 1j). As shown in the sketch in Figure 1b, the SLD contrast and thus the SANS intensity increase when these components are formed (Table S1 shows SLD values for carbon, Li_2_S, and possible CEI components). The fact that the SANS intensity curve has a similar shape to the SAXS intensity of the empty nanopore structure (Figure 1a) indicates that Li_2_S and CEI components are formed inside the nanopores and not only on the outer surface of the AC08 particles (as shown by the surface-sensitive XPS data in ref (33)). The SANS intensities remain nearly identical during further charging (Figure 1e) and discharging (Figure 1f). Hence, the solid components in the nanopores remain largely intact and are not dissolved during electrochemical conversion (Figure 1j). The slight *q*-shift of the SANS intensity during solid-state conversion (Figure 1e,f) might be attributed to the detailed SLD change and swelling/shrinking of the active materials inside the pores or the pore structure itself upon lithiation/delithiation.^45,46^ Also, the pore structure itself is expected to show some degree of swelling/shrinking during lithiation/delithiation. However, the effect on the SANS and XRD intensities is minimal and does not impact the conclusions drawn from the data. If carbon swelling were substantial during cycling, we would expect noticeable changes between 20° and 30° around the carbon 002 peak.

The *operando* XRD measurements in Figure 1g–i are also consistent with the solid-state S/Li_2_S conversion occurring inside the AC pores. Figures S7–S8 show the reference data of pristine AC, S, AC-S powder, and the AC electrode impregnated with sulfur. Orthorhombic S_8_ crystals exhibit sharp diffraction peaks (Figure S8c), whereas the electrode after sulfur impregnation shows only a broad peak between 10 and 30° 2θ and no distinct peaks that would indicate bulk sulfur crystals. The broad peak is likely caused by amorphous or nanocrystalline S clusters inside the carbon nanopores, as the Scherrer crystallite size is in the order of 0.8–1.0 nm (Figure 1g–i, red curves, and Figure S7). TGA measurements of the pristine AC08/S powder at C/S 1/1 confirm that essentially all S is present inside the nanopores (Figure S1). Comparable to the pristine AC08-S electrode, the XRD patterns after discharge and charge show a broad peak between 10° and 30° 2θ. This indicates that no bulk crystalline phase is formed outside the nanopores throughout cycling and that sulfur and other discharge products (Li_2_S and Li_2_S_*x*_) are present in their amorphous state,^47^ most likely in the nanopores.

SEM of the AC08-S electrode in its pristine state, after the first discharge and after the first charge, reveals the formation of cracks and some surface deposits,^48,49^ which are likely CEI components (Figure S9).^35^ Particle fracture after the first discharge is likely caused by the formation of CEI components and Li_2_S inside the nanopores.^5,46,50,51^

**Figure 2:**
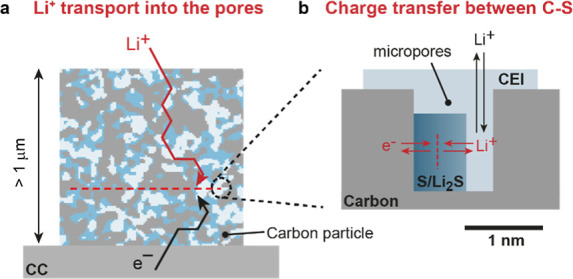
Possible rate and capacity limiting factors during solid-state conversion in confinement. Both (a) Li ion transport into the carbon particle and (b) charge transfer between carbon and active material could be rate-limiting factors.

In summary, *operando* SANS and *operando* XRD measurements provide evidence that the solid-state S/Li_2_S conversion occurs inside the carbon nanopores and that the CEI also exists within the nanopore network (Figure 1j). Using electrochemical measurements, we explore two factors that could potentially limit the cathode’s rate performance and capacity (Figure 2): (1) Li-ion (mass) transport into and in the carbon particles (Figure 2a) and (2) charge transfer between the active material and the electronically conductive carbon (Figure 2b).

### Electrochemical Processes

Electrochemical processes in a full-cell Li–S battery were monitored with GITT-EIS during galvanostatic charging and discharging between 0.5 V and 3 V vs Li/Li^+^ with a rate of C/10. Every 100 (200 for the first discharge) mA h g_S_^–1^, the cells rest for 1 h at OCV, at which point EIS is performed. Full cell impedance measurements are rich in information and contain features from both the cathode and the anode. Developing a quantitative equivalent circuit model in which each element has a physicochemical origin is difficult. Here, we compare full-cell EIS/GITT data of two cells with two different nanoporous carbons, AC08 and AC12, with a mean nanopore size of 0.8 and 1.2 nm, but otherwise identical cells components. This helps us to identify impedance features that are linked to the nanopore structure of the cathode particles. Identifying trends during charging and discharging can hint at the origin of certain features. The complete data set of EIS/GITT measurements over the entire frequency range is given in Figure S10 (AC08) and Figure S11 (AC12).

The impedance spectrum of a symmetric cell consisting of two cathodes after the first discharge, first charge, and second discharge shows that Li metal impedance does contribute to the mid-frequency range features (10–1000 Hz) of the full cell. However, the symmetric cell results also show that this contribution is small and that the full cell impedance is dominated by processes in the cathode (Figure S12).

**Figure 3:**
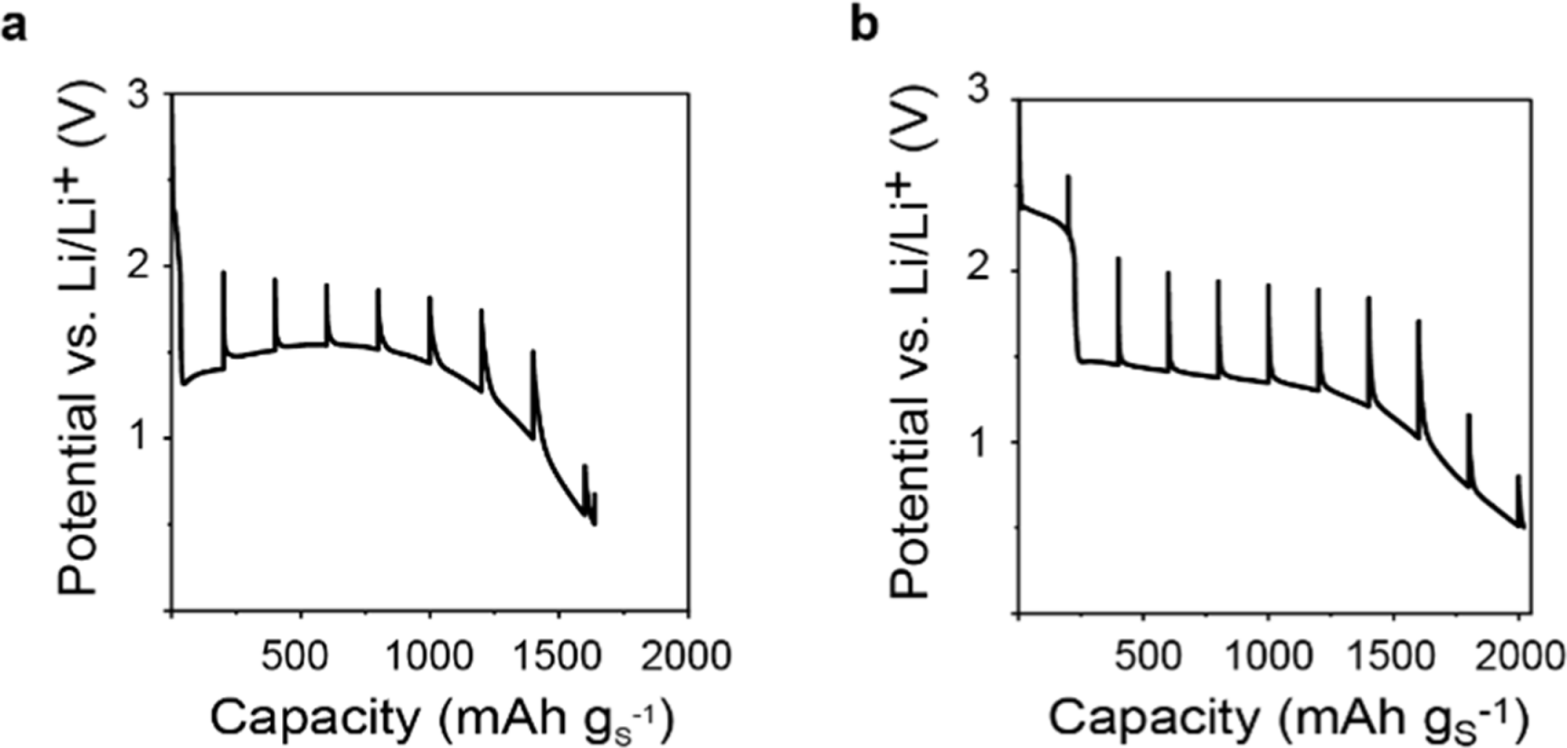
Galvanostatic intermittent titration technique (GITT). GITT measurements during the first discharge at a rate of C/10 for (a) AC08/S (C/S 1/1) and (b) AC12/S (C/S 1/1) electrodes.

The first discharge curves (Figure 3a,b, Figure S10a, and Figure S11a) show two potential plateaus. The first plateau at ∼2.3 V vs Li/Li^+^ indicates the reduction of S to dissolved long-chain polysulfides.^10,52^ This plateau is shorter in the smaller-pored carbon (Figure 3a) than in the large-pored carbon (Figure 3b), indicating that more S is reduced to polysulfides in the larger carbon pores. However, the plateau for both nanoporous carbons is short compared to that observed in ether-based systems,^7^ indicating that impregnating S in nanopores reduces the amount of S reduced to long-chain polysulfides.

The second part of the plateau is a ∼1.5 V vs Li/Li^+^, with the equilibrium voltage reached during OCV ∼2.0 V vs Li/Li^+^, pointing to solid-state Li_2_S formation^5^ (in ether-based electrolytes, a plateau at 2.15 V vs Li/Li^+^ is common^53^). The overpotentials are high for both carbons (0.4 V–0.7 V). Lower overpotentials after the CEI formation in further cycles suggest that the large overpotential could be related to dissolved polysulfides and their reactions with the carbonate solvent and the formation of CEI components.^54^ Notably, the first discharge profiles for AC08 and AC12 differ: AC12 displays a relatively flat plateau near 1.5 V, while AC08 exhibits a marked curvature. Given that equilibrium potentials are similar for both, this curvature in AC08 is likely attributable to distinct kinetic properties arising from its nanopore structure and particle morphology, possibly introducing a mass transport limitation for Li ions or polysulfides. This interpretation is complemented by EIS data (Figure S10a–c and Figure S11a–c and further discussion in Supporting Information Note 1).

**Figure 4:**
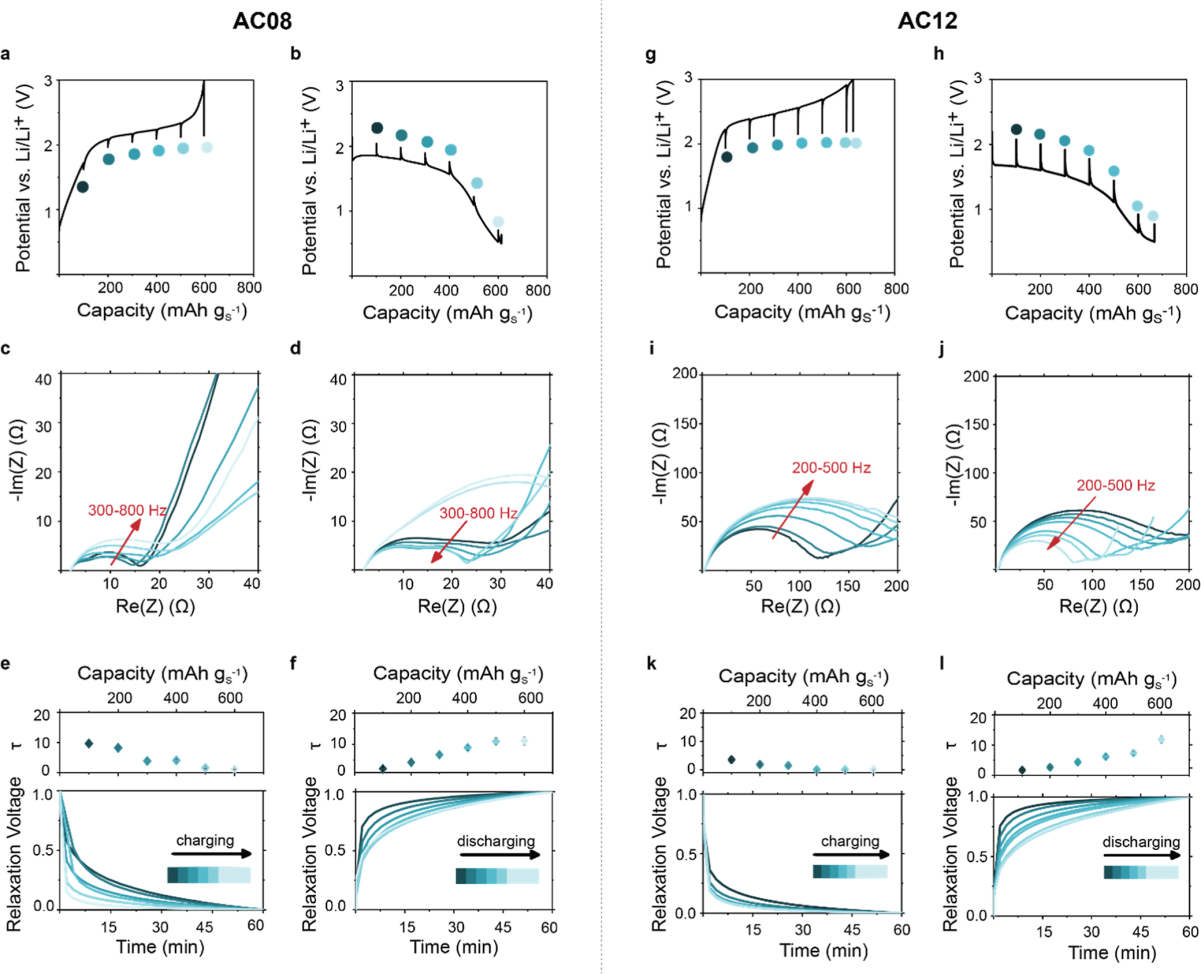
In situ EIS and relaxation voltage data of AC08/S and AC12/S during GITT measurements. (a,b) GITT curve during the first charge (a) and second discharge (b) for the AC08/S (C/S 1/1) electrode at C/10 and RT. The dots represent the EIS measurement points; colors match the spectra and relaxation times shown in (c–f). (c) EIS Nyquist plots in the high and mid-frequency region during the first charge for AC08/S. (d) EIS Nyquist plots in the high and mid-frequency region during the second discharge for AC08/S. (e,f) Normalized relaxation voltage of the OCV period during GITT measurements for the AC08/S during the first charge/delithiation (e) and second discharge/lithiation (f). (g,h) GITT curve during the first charge (g) and second discharge (h) for the AC12/S (C/S 1/1) electrode at C/10 and RT. The dots represent the EIS measurement points; colors match the spectra and relaxation times shown in (i–l). (i) EIS Nyquist plots in the high and mid-frequency region during the first charge for AC12/S. (j) EIS Nyquist plots in the high and mid-frequency region during the second discharge for AC12/S. (k,l) Normalized relaxation voltage of the OCV period during GITT measurements for the AC12/S during the first charge/delithiation (k) and second discharge/lithiation (l).

Figure 4 shows the EIS/GITT results for solid-state conversion after the CEI has been formed. Results for AC08 are shown in Figure 4a–f and those for AC12 in Figure 4g–l. During charging (Figure 4a,g) and discharging (Figure 4b,h), there are single potential plateaus around 2.0 V vs Li/Li^+^ and 1.8 V vs Li/Li^+^, confirming that after the initial formation of the CEI, there is no further dissolution of S to polysulfides, and solid-state S/Li_2_S conversion dominates.^5^ The overpotentials decrease at the end of the discharge, indicating that the final slope reflects a specific capacitive contribution, which is reversible at the beginning of charging. In the EIS, we will focus on the mid-frequency region (10–1000 Hz), as it shows the most distinct difference between AC08 and AC12. A distinct arc emerges between 200 and 700 Hz, with resistance increasing during charge and decreasing during discharge for both materials. AC12, with larger nanopores, shows resistance values 6–10 times higher than AC08. It is difficult to determine the origin of this feature; we speculate that the increase in resistance values with larger nanopores may suggest some form of increased charge transfer resistance. If the Li metal impedance would dominate the mid-frequency full cell impedance, we would expect opposite trends with charging and discharging (see symmetric anode–anode tests in Figure S12e,f). The Bode plots in Figures S13 and S14 reveal the same trends in the mid-frequency region.

The time-dependent potential relaxation during OCV (normalized and shown in Figure 4e,f–k,l) reveals decreasing relaxation time constants τ during charging and increasing times during discharge for both materials (consistent with ref (38)), with overall shorter relaxation time constants for AC12. Lower relaxation times with larger nanopores may suggest Li-ion diffusion in and out of the particle to be a dominating factor,^55^ even though slower processes of another origin may also contribute.

In the following, we systematically vary material parameters and battery testing protocols to understand how processes like charge transfer resistance or effective Li-ion transport in and out of the particles could affect rate performance and capacities.

### Electrochemical Performance

**Figure 5:**
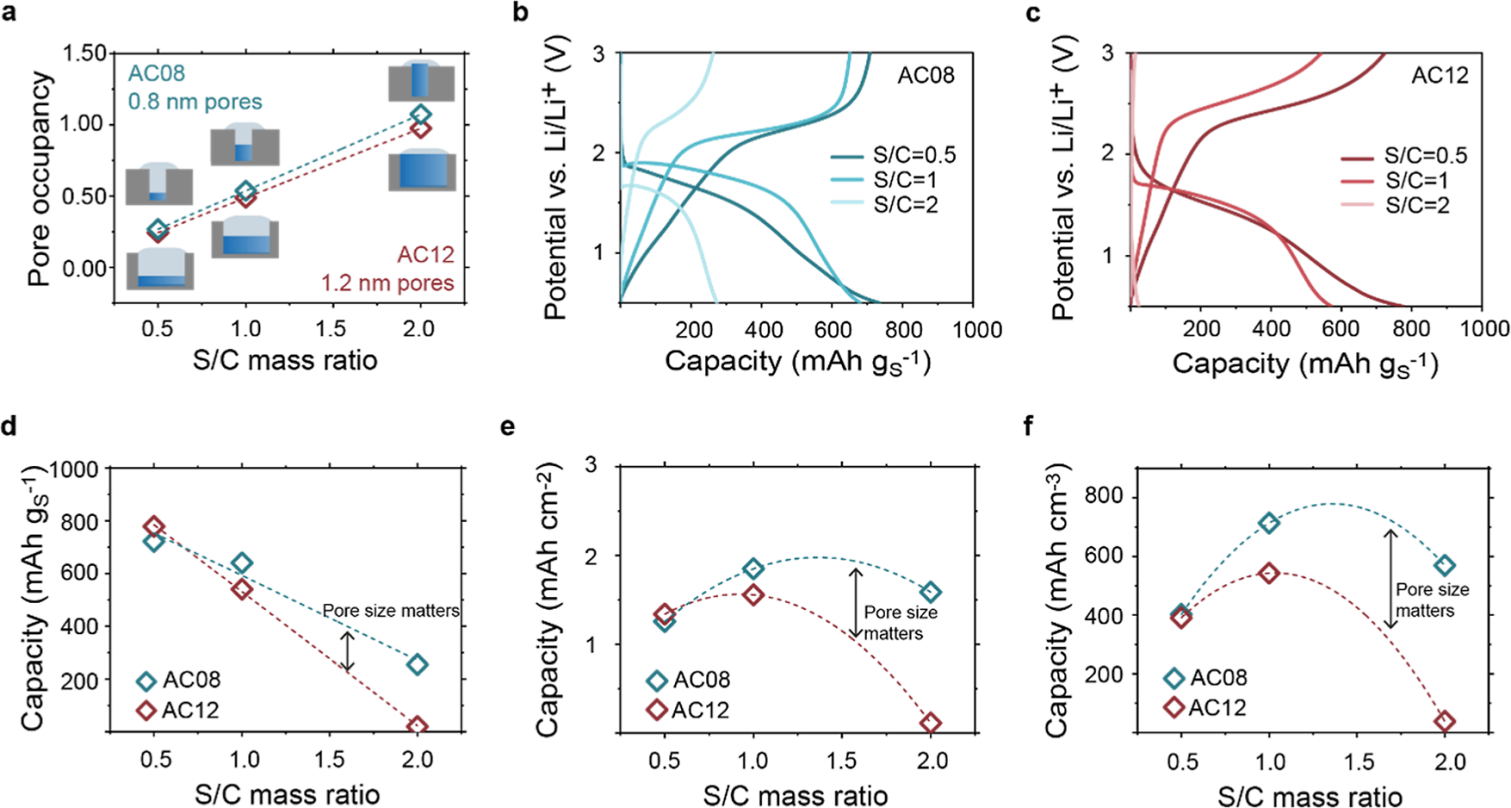
Effect of carbon pore size and sulfur loading on the electrochemical performance. (a) Theoretical pore occupancies as a function of S/C mass ratio. The dotted line serves as a guide to the eye to represent the linear relation. (b,c) Galvanostatic charge/discharge of AC08 (b) and AC12 (c) with different sulfur/carbon ratios (third cycle displayed). (d) Specific capacities (discharge at the fifth cycle) as a function of the sulfur/carbon mass ratio. The dotted line (linear fit) serves as a guide to the eye. (e) Capacity loading in mAh cm^–2^ (discharge at the fifth cycle) as a function of the sulfur to carbon mass ratio. The dotted curve (polynomial order of 2) serves as a guide to the eye. (f) Capacity loading in mA h cm^–3^ (per pore volume of pristine carbon based on GSA measurements (discharge capacity at the fifth cycle) as a function of the sulfur to carbon mass ratio. The dotted curve (polynomial order of 2) serves as a guide to the eye.

We tested the two activated carbons with similar surface areas but different mean pore sizes (AC08 and AC12) and three different carbon-to-sulfur (C/S) mass ratios: C/S 2/1, C/S 1/1, and C/S 1/2. Because the total pore volume is similar for both carbons, the theoretical pore volume occupancy for the different S loadings is the same for the two carbons, where the 1/1 S/C mass ratio would theoretically enable approximately 50% of pore volume to be occupied (Figure 5a). Thermogravimetric analysis (Figure S1) shows that in the pristine samples with lower (C/S 2/1) and intermediate (C/S 1/1) sulfur fractions, essentially all sulfur is present inside the nanopores. For the highest sulfur fraction (C/S 1/2), a certain fraction of sulfur exists outside the nanopores as surface sulfur. The precise amount of active material retained within the nanopores after CEI formation remains uncertain. The cells were cycled between 0.5 and 3 V with a C/10 cycling rate. The third charge and discharge cycles are shown for AC08 (Figure 5b) and AC12 (Figure 5c). The single plateau in the charge/discharge processes confirms solid-state conversion for all samples; however, the electrochemical performance depends strongly on the pore size and S loading. The overpotential is the smallest for the C/S ratio of 1/1 and increases with increasing pore size.

For both carbons, the capacity per gram of sulfur (specific capacity) decreases with increasing S/C content (Figure 5d). This indicates that the fraction of S that converts decreases with increasing S mass loading. This decrease is more pronounced for the larger pores. Also, with an increasing S content, the discharge capacity decays more drastically in larger pores (Figure S15). This implies there is an optimum S/C ratio. Indeed, the capacity loading (mA h cm^–2^, capacity per geometrical cathode area) and the capacity per pore volume (mA h cm^–3^) are highest for a S/C ratio of around 1/1 (Figure 5e,f). Smaller nanopores also show superior per-area performance in the long term (Figure S16).

This data suggests that

(i) Partial filling of pores is favorable. It may allow the formation of CEI inside the nanopores and the accommodation of the volume expansion during lithiation.

(ii) More small pores are favorable compared to fewer large pores. Assuming lithium diffusion is favored in larger pores, charge transfer between the S/Li_2_S and the carbon network limits the conversion. Solvent accessibility in the nanopores may determine the active material/CEI structure and thus lead to a larger fraction of inactive sulfur in carbons with larger nanopores.

(iii) Capacity is limited to a significant extent by charge transfer and not primarily by Li-ion diffusion into and out of the nanoporous carbon. If Li-ion diffusion were limiting, we would anticipate the lowest overpotentials at the lowest loadings of sulfur (S/C) and in the larger pores.

**Figure 6:**
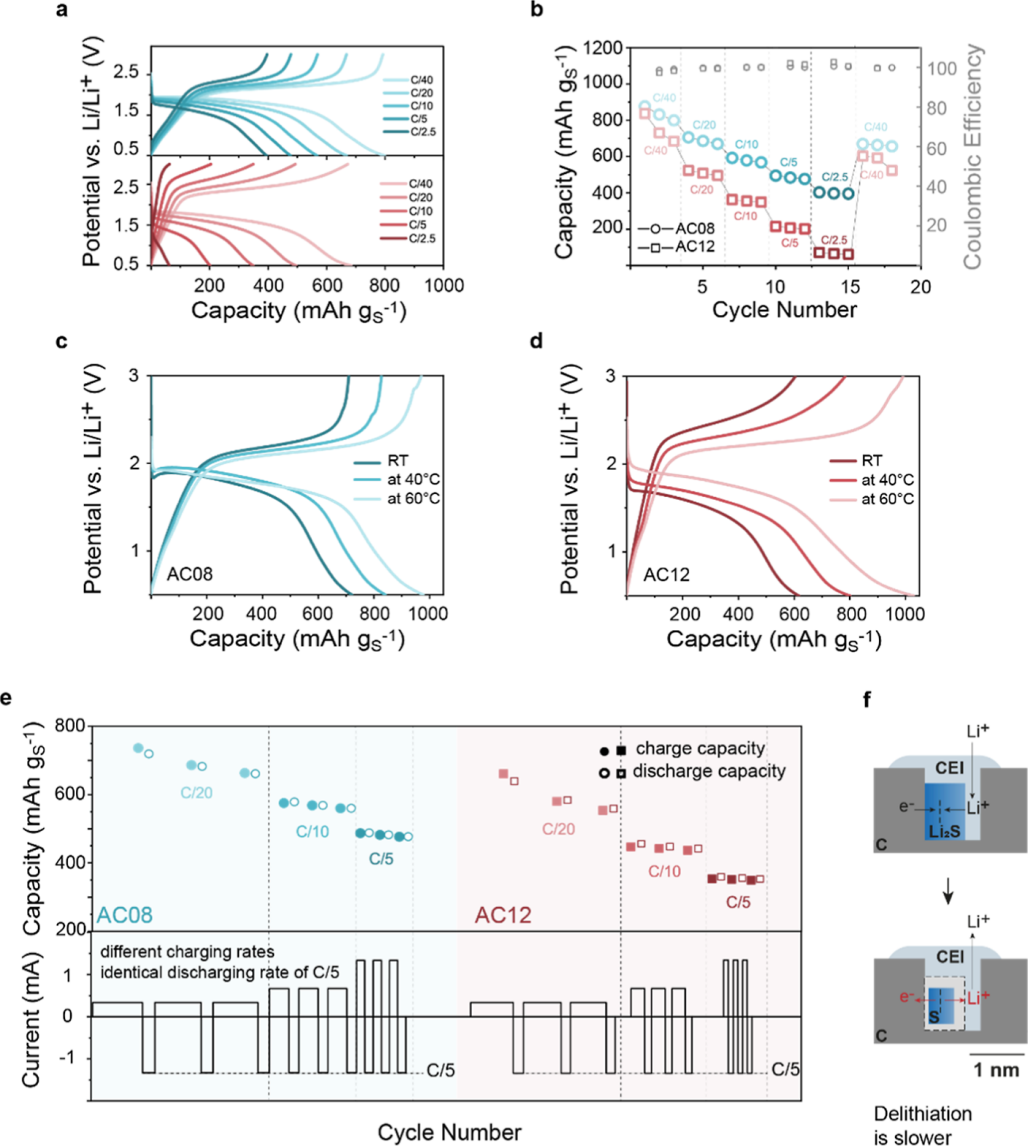
Rate capability data based on gravimetric capacities. (a) Galvanostatic (dis)charge curves with different (dis)charge rates indicated as color tones. Blue and red represent the pore size of 0.8 nm (AC08) and 1.2 nm (AC12), respectively. The sulfur loading of the cathodes is 4.23 mg_s_ (AC08) and 4.44 mg_s_ (AC12) at a C/S mass ratio of 1/1. (b) Discharge capacities (left axis) and Coulombic efficiencies (right axis) at different rates of C/40, C/20, C/10, C/5, and C/2.5. (c,d) Galvanostatic (dis)charge curves with different temperatures (RT, 40 °C and 60 °C) at C/10 for AC08 (c) and AC12 (d) indicated as color tones. (e) Specific capacities for constant discharge rates but varying charge rates for AC08 and AC12. All cells are discharged with C/5, the charge rates are varied from C/20 to C/5. Filled shapes correspond to charging capacities, and unfilled shapes correspond to discharge capacities. (f) Illustrative representation of conversion during cycling. Volume changes during cycling and especially shrinking during charging could be critical for the rate performance.

The rate capability is tested with discharging/charging rates of C/40, C/20, C/10, C/5, and C/2.5 for the two C–S composite cathodes (AC08 and AC12) and a C/S ratio of 1/1 (Figure 6a). We find that a higher current density results in a larger overpotential and a lower specific capacity (i.e., a lower sulfur utilization).^56^ At all rates, more capacity can be extracted with smaller pores (AC08), but this becomes more pronounced at higher rates (Figure 6b). At C/2.5, a capacity of around 400 mA h g_S_^–1^ is possible for AC08, while the capacity is below 50 mA h g_S_^–1^ for the larger pore carbon AC12. An explanation for this dependency could be the improved contact between sulfur and carbon in smaller pores, reducing the charge transfer resistance.^56,57^

Even at C/20, there is no full conversion of S as indicated by the fact that the capacity can be further increased when the rate is decreased to C/40. We also observe that the capacity decay in Figure 6b is most prominent at slower rates, indicating that the capacity fade is related to the time of reaction, e.g., self-discharge, some degree of polysulfide shuttling or side reactions at the anode. Figure S17 shows additional rate performance tests at rates from C/10 to 1C with more cycles per rate. The data confirm the results in Figure 6b.

Also, the temperature dependence specific capacities underline the kinetic limitation of S/Li_2_S conversion. With an increase in temperature, the specific capacity generally increases (Figure 6c,d and Figure S18).

We then investigated whether the charging or discharging is the rate-determining step by discharging with C/5 but charging with C/20, C/10, and C/5. For both carbons, the discharge and charge capacities are nearly identical for a given specific charging rate and decrease as the charging rate is increased (Figure 6e). When the charging rate is increased, there is a clear increase in the overpotential (Figure S19). This means that the discharge yield is controlled by the charging rate and that delithiation is slow compared to lithiation.

A possible explanation for the capacity being limited by the charging rate is that during charging, the active material shrinks, which could allow faster Li-ion transport (Figure 4) but hinder the charge transfer between carbon and active material (Figure 6f). Charge transfer between the carbon/active material interface likely contributes significantly to the rate limitation and is critical, especially during charging.

## Conclusions

(i) Operando SANS confirms CEI formation during the first discharge, also inside the nanopores and not only on the outer surface of particles. Hence, nanopores are filled with CEI components, such as LiF, during the first discharge. The formed CEI/active material structure remains stable during further solid-state conversion. Operando XRD indicates that active materials (S and Li_2_S) remain amorphous at all times for further solid-state conversion.

(ii) EIS/GITT results show distinct differences between the two carbons, which are attributed to variations in their nanopore structure: AC12, with its larger nanopores, shows a more pronounced mid-frequency impedance arc, which increases in resistance during charging and decreases during discharging. Relaxation times at OCV, often linked to effective Li-ion diffusion, decrease during charging and increase during discharging and are generally lower for AC12 with larger nanopores.

(iii) Galvanostatic cycling tests indicate improved performance and lowest overpotentials for a C/S fraction of 1/1. Reducing or increasing the sulfur fractions increases the overpotentials. Carbons with smaller nanopores exhibit higher capacities and improved rate performance. Results also suggest that capacity is primarily governed by the charging step; while discharging can occur at relatively high rates, charging is the limiting factor for cell rate performance.

These findings indicate stable CEI formation during the first cycle and solid-state S/Li_2_S conversion inside the nanopores of a sulfur-infused, activated carbon cathode. Conversion occurs optimally when pores are narrow and filled, probably due to enhanced charge transfer across the active material–carbon interface in confined spaces. Charge transfer appears to be an important factor limiting the capacity, especially during charging. We speculate that a low number of intimate contact points between the active material and carbon leads to high charge transfer resistance. Carbons with subnanometer pores also incorporate fewer solvent molecules, leading to a lower fraction of CEI and a higher fraction of S/Li_2_S in close proximity to the carbon surface, hence improving charge transfer rates.

These insights into solid-state S/Li_2_S conversion in S-impregnated carbon suggest that relatively high-performance Li–S batteries can be achieved with a carbonate electrolyte by creating a multiscale carbon structure that enables fast solid-state lithium diffusion and high-rate solid-state S/Li_2_S conversion. The former is achieved by small particle size (e.g., 1 μm), and the latter by a nanopore structure with a large fraction of narrow pores smaller than about 0.8 nm. Our work demonstrates that the nanopore structure is the more significant parameter for the solid-state conversion kinetics, at least in AC particles not larger than 20 μm. Nanoporous carbons with catalytically active surface groups like nitrogen could further improve the bonding between the carbon substrate and S and thus reduce the charge transfer resistance.

A key factor that requires further study is the formation of the CEI/Li_2_S structure during the first discharge and the quantity of active material inside the nanopores after CEI formation. CEI formation involves dissolved polysulfides potentially leaching out before CEI formation, which decisively influences the quantity and distribution of active material within carbon particles. In addition to the nanopore structure, particle size and mass transport may also significantly affect CEI/Li_2_S formation during the first discharge and the amount of active material available for subsequent confined solid-state conversion.

## Data Availability

All experimental (raw-)data of this study are available under https://doi.org/10.5281/zenodo.14048781.

## Supporting Information Available

TGA measurement results of C–S powders; N_2_ adsorption measurement results of C–S; sketches of the operando scattering and diffraction setups and operando cells; primary SANS data and background subtraction procedure; galvanostatic charge/discharge data of the in situ XRD measurements; ex situ XRD measurements of pure carbons, sulfur, C–S powders, and C–S electrodes; scanning electron micrographs of the pristine electrode, electrode after the first discharge and the electrode after the first charge; in situ EIS and relaxation voltage analysis of the cells and further discussion; symmetric cell impedance measurements; Bode plots; galvanostatic performance of cells with different sulfur loadings and carbon types; long-term cycling and Coulombic efficiency data; rate performance; galvanostatic performance of cells at different temperatures; charge–discharge curves of the rate performance tests; SLDs of carbon, sulfur, lithium sulfide, and possible CEI components; and supporting note on impedance data (PDF)
