# Supplementary material for: Understanding Rate and Capacity Limitations in Li–S Batteries Based on Solid-State Sulfur Conversion in Confinement
Source: ACS Appl Mater Interfaces. 2024 Nov 29;16(49):67651–61. doi: 10.1021/acsami.4c13183 (PMC11647752; doi:10.1021/acsami.4c13183)
Supplement: Supplementary file 1 — am4c13183_si_001.pdf [file am4c13183_si_001.pdf]

Supporting Information

Understanding Rate and Capacity Limitations in Li-S Batteries based on Solid-state Sulfur Conversion in Confinement

Ayca Senol Gungor[1], Jean-Marc von Mentlen[1], Jean G. A. Ruthes[2,3], Francisco J. García-Soriano[4], Sara Drvarič Talian[4], Volker Presser[2,3,5], Lionel Porcar[6], Alen Vizintin[4], Vanessa Wood[1,*], Christian Prehal[1,7,*]

1 Department of Information Technology and Electrical Engineering, ETH Zürich, Gloriastrasse 35, 8092 Zürich, Switzerland

2 INM - Leibniz Institute for New Materials, Campus D2 2, 66123 Saarbrücken, Germany

3 Department of Materials Science and Engineering, Saarland University, Campus D2 2, 66123 Saarbrücken, Germany

4 Department of Materials Chemistry, National Institute of Chemistry, Hajdrihova 19, 1000 Ljubljana, Slovenia

5 saarene – Saarland Center for Energy Materials and Sustainability, Campus C4 2, 66123 Saarbrücken, Germany

6 Institut Laue–Langevin, 71 Avenue des Martyrs, Grenoble, 38042, France

7 Department of Chemistry and Physics of Materials, Paris-Lodron University of Salzburg, Jakob-Haringer-Straße 2a, 5020 Salzburg, Austria

Corresponding authors' email: christian.prehal@plus.ac.at, vwood@ethz.ch

1. Supporting Figures

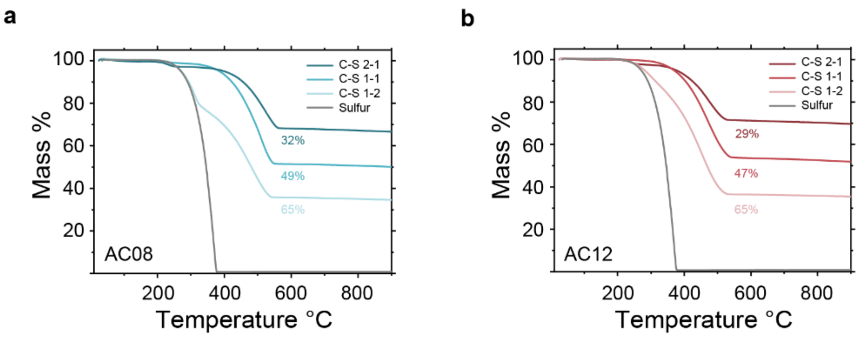

Figure S1: TGA measurement results of with (a) AC08 (b) AC12 powders with different sulfur loadings.

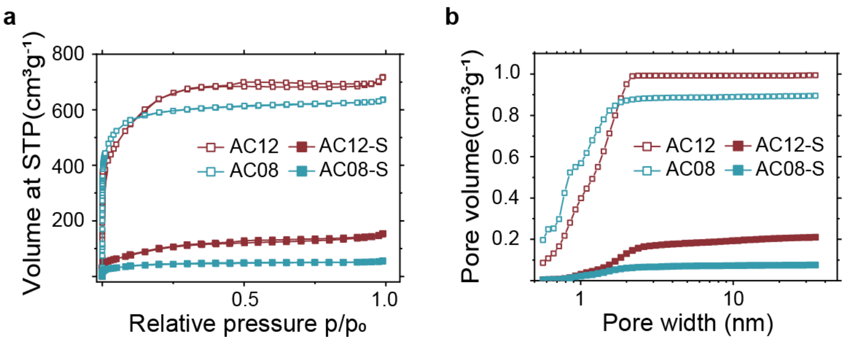

Figure S2: N_2 adsorption measurement results of AC08 and AC12 with the C-S mass loading of 1/1. (a), Volume at STP with respect to the relative pressure. (b), Pore volume with respect to pore width.

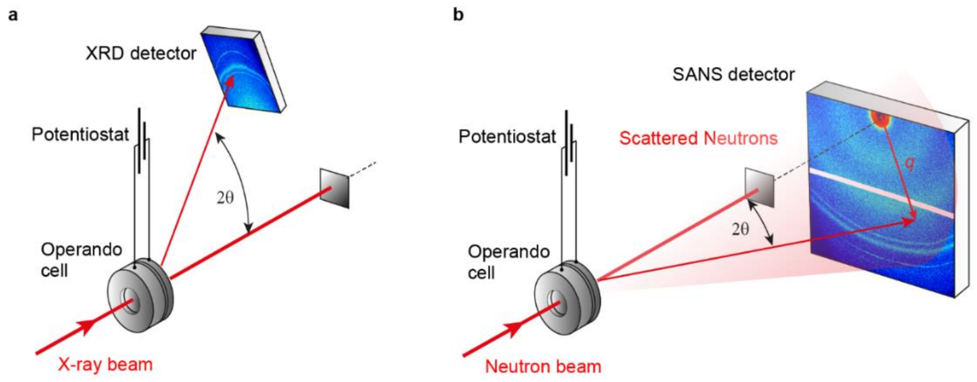

Figure S3: Sketch of the operando experimental set-up. (a), Set-up for operando X-ray diffraction (XRD). (b), Set-up for operando small angle neutron scattering (SANS).

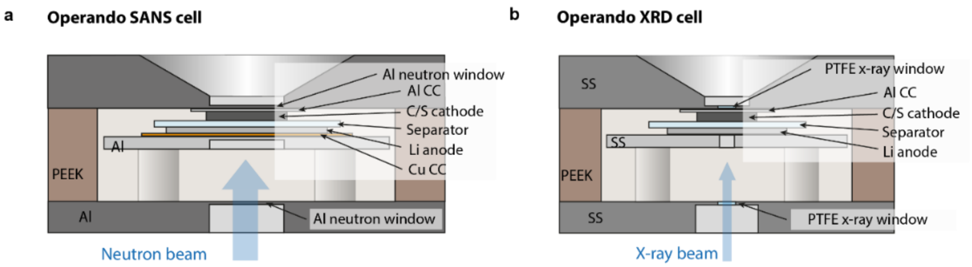

Figure S4: Schematics of the operando SANS and XRD cells. SANS (a) and XRD (b) cells have similar designs, although aluminum windows of SANS cells are replaced with thin PTFE windows.

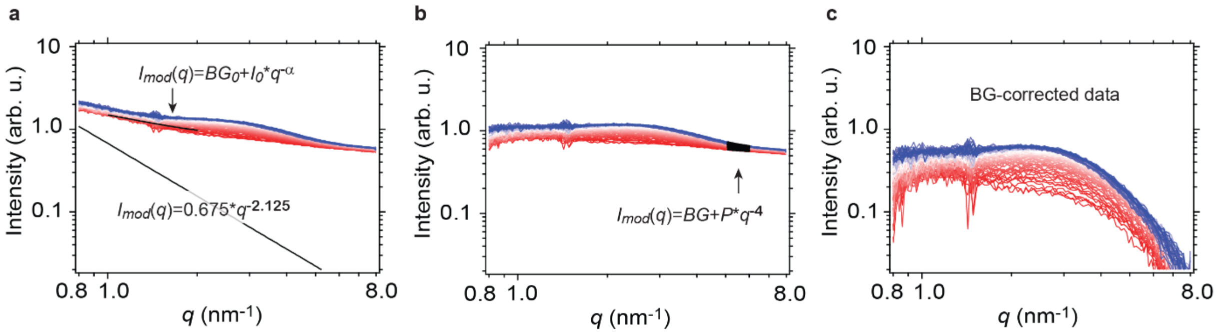

Figure S5: Primary SANS data and background subtraction procedure. (a), Operando SANS intensities vs. scattering vector length *q* during galvanostatic charge/discharge (see Figure 1, main part), after transmission correction and before background subtraction. Red curves correspond to the beginning of the first discharge, blue curves to the end of the second discharge. The black curve shows the power law model fitted to the SANS intensity right after the high-voltage plateau during the first discharge has ended. The fit range lies between 1 and 2 nm^{-1}. The resulting power law curve (bottom black curve) is then subtracted (b), SANS intensities vs. *q* after subtracting the low-q power law curve. The constant background originating from incoherent/diffuse scattering is then determined with a Porod-fit in a range from 5 – 6 nm^{-1} (black curves). (c), SANS intensities vs. *q* after subtracting the constant background determined with the Porod fit in (b).

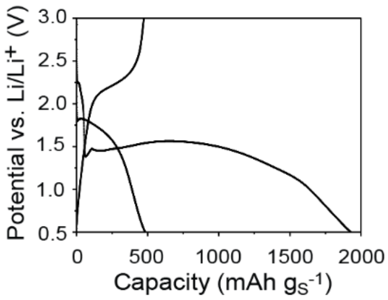

Figure S6: Galvanostatic charge/discharge data of the in-situ XRD measurements. Electrode potential vs. Li/Li^+ as a function of the specific capacity of AC08-S 1-1 electrode.

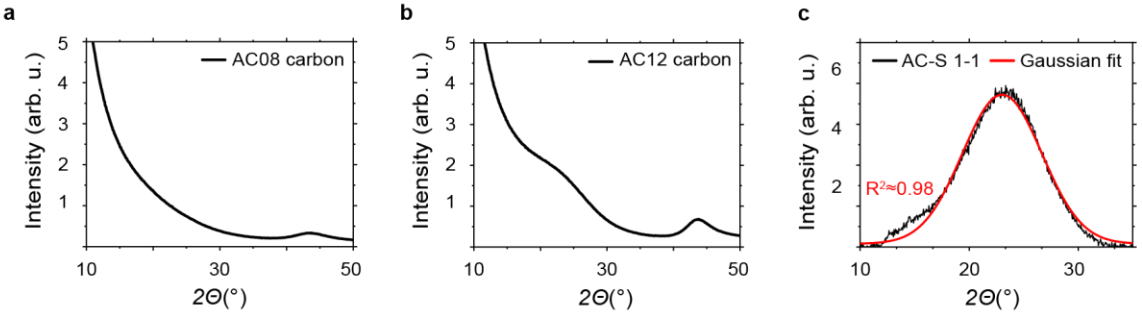

Figure S7: Ex-situ XRD measurements of pure AC08 (a), AC12 (b) carbon and AC08-S (c) carbon-sulfur powders. All data is recorded in reflection mode. (c) From the Gaussian fit to the pristine AC08-S carbon (R^2≈0.988), the width of the Gaussian distribution (FWHM) is calculated. The crystallite size is calculated then around 1.04 nm by the Scherrer equation and FWHM.

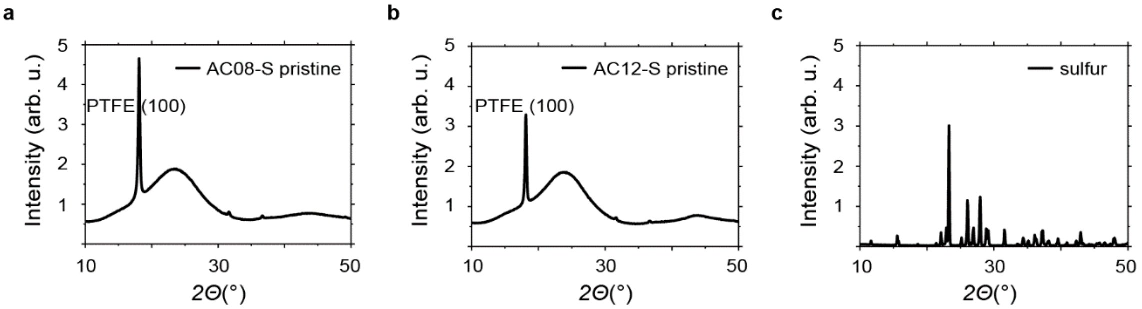

Figure S8: Ex-situ XRD measurements of pristine AC08 electrode (a), pristine AC12 electrode (b) and sulfur powder (c). All data is recorded in reflection mode. There is no sulfur peak recorded in the pristine electrodes.

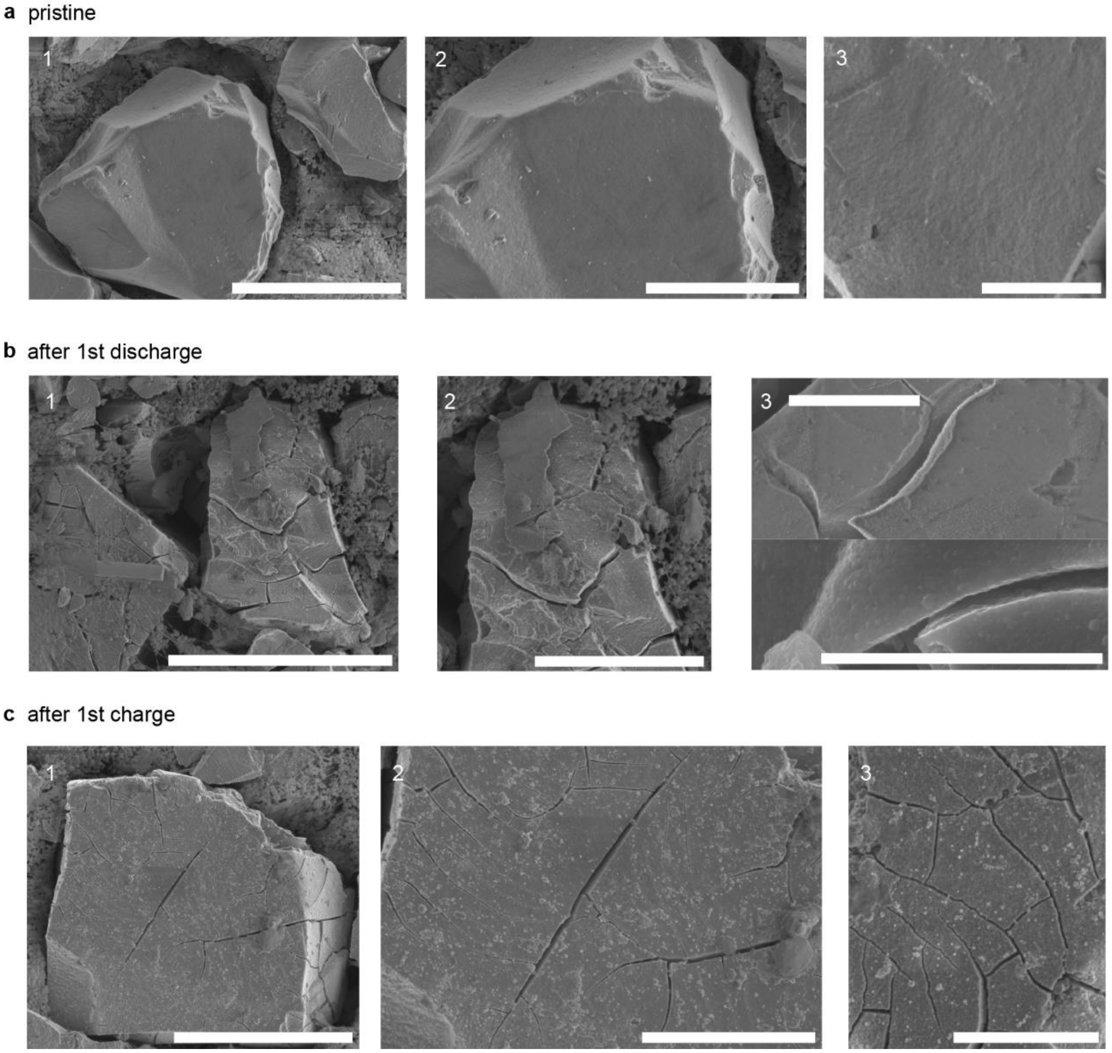

Figure S9: Scanning electron micrographs of (a) the pristine electrode (b) the electrode after the first discharge and (c) the electrode after the first charge. Scale bars represent 10 µm in micrographs (1), 5 µm in micrographs (2) and 2 µm in micrographs (3). (a) The pristine activated carbon with an approximate size of 10-12 µm (1) and the zoomed in. As seen in micrograph (3) the particle surface is smooth and the particle is intact. (b) Activated carbon particles after the first discharge. The particles show cracks because of the volume expansion during CEI and Li_2S formation and the stress on the carbon backbone. Deposits on the surface of the carbon particles might be related to CEI components. (c) Activated carbon particles after the first charge. The cracks remain after the active material volume decrease during de-lithiation.

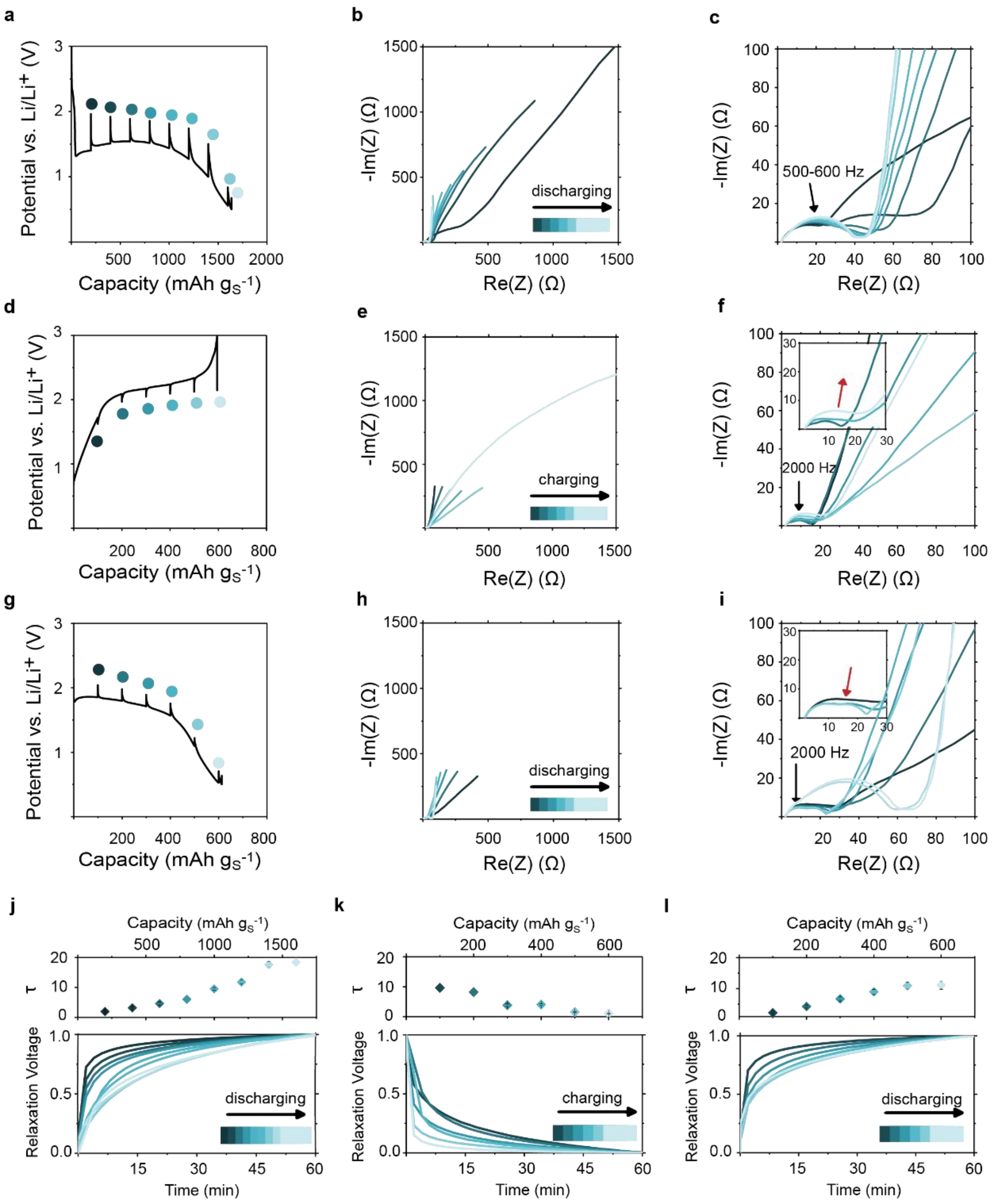

Figure S10: ***In-situ* EIS and relaxation voltage data of AC08 during GITT measurements.** (a) Galvanostatic intermittent titration technique (GITT) curve during the first discharge. The dots represent the EIS measurement points; colors match the spectra shown in (b) and (c). (b) EIS Nyquist plots during the first discharge at RT. (c) Magnification of the high and mid-frequency region of the data shown in (b) (more detailed discussion in Supporting Note 1). (d) GITT curve during the first charge. (e) EIS Nyquist plots of the first charge. (f) Magnified EIS Nyquist plots, representing the high and middle frequency regions. The impedance response indicated at high frequencies (2000 Hz) might be related to Li metal anode. (g) GITT curve during the second discharge. (h) EIS Nyquist plots during the second discharge. (i) Magnification of the high- and middle-frequency region of the data shown in (h). At high frequencies, the traces of Li anode are seen. Normalized Relaxation voltage of the open circuit voltage period during GITT measurements during first discharge (lithiation) j), first charge (delithiation) (k) and second discharge (lithiation) (l).

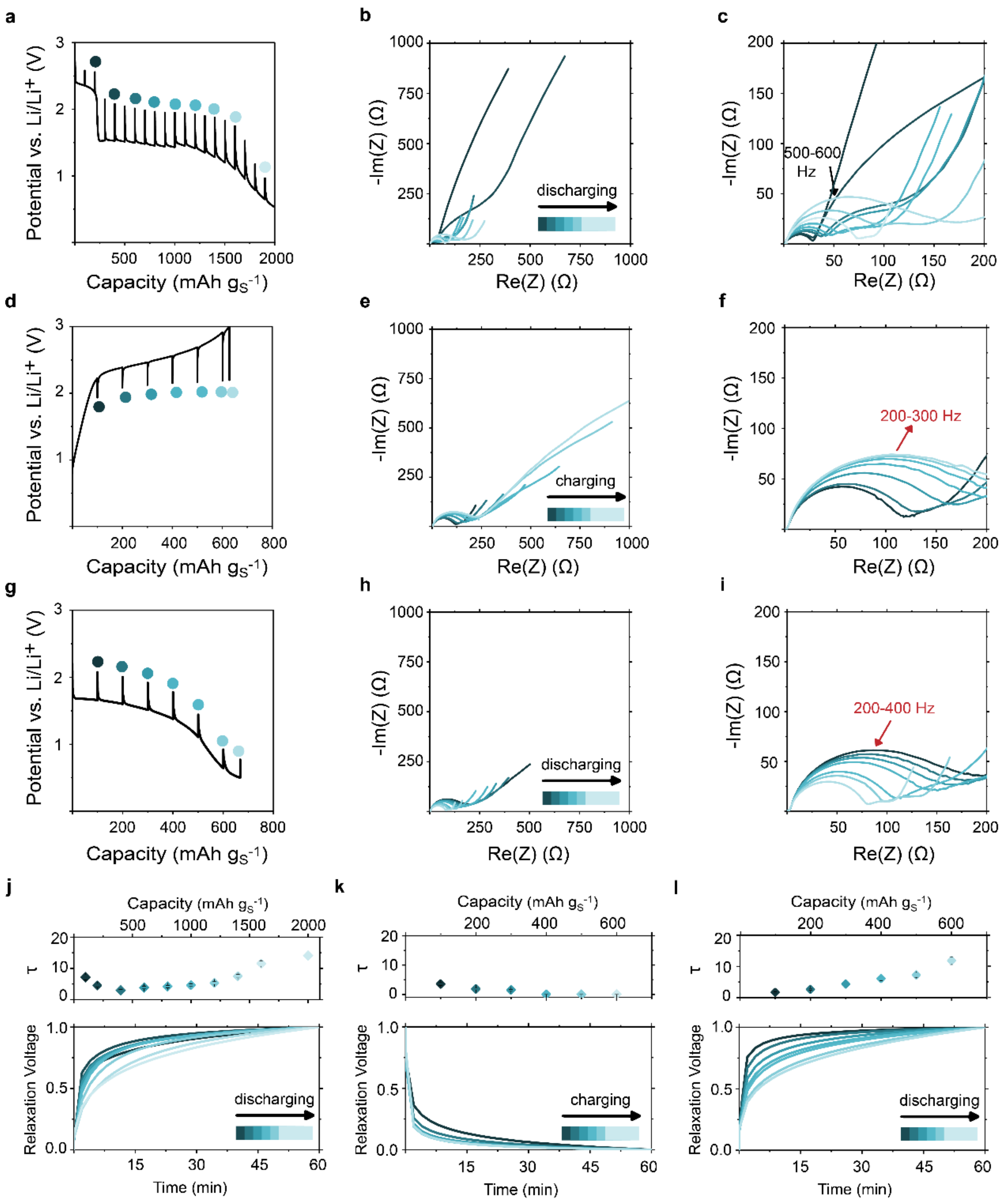

Figure S11: **Operando EIS data of AC12/S (C/S mass ratio of 1/1) during GITT measurements.** (a) EIS Nyquist plots during the first discharge at room temperature. (b) Magnification of the high and middle frequency region of the data shown in (a). (c) Galvanostatic intermittent titration technique (GITT) curve during the first discharge. The dots represent the EIS measurement points; colors match the spectra shown in (a) and (b). The impedance change during discharge corresponds to the CEI formation and electrochemical Li_2S formation. (d) EIS Nyquist plots of the first charge. (e) Magnified EIS Nyquist plots, representing the high and middle frequency regions. (f) GITT curve during the first charge. (g) EIS Nyquist plots during the second discharge. (h) Magnification of the high and middle frequency region of the data shown in (g). (i) GITT curve during the second discharge. Normalized Relaxation voltage of the open circuit voltage period during GITT measurements during first discharge (j), first charge (k) and second discharge (l).

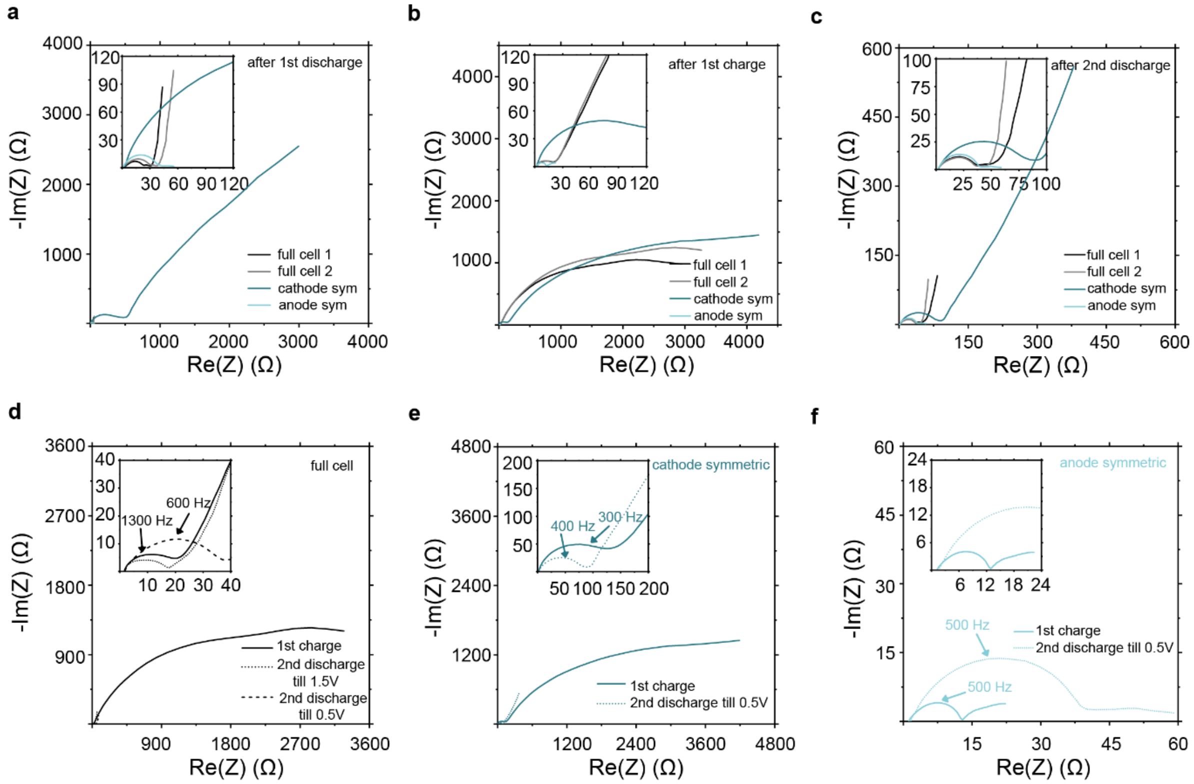

Figure S12: Symmetric cell impedance measurement of the AC08-S electrodes (C/S mass ratio of 1/1) during first discharge (a), first charge (b), and second discharge (c). The symmetric cell impedance responses determine whether an EIS feature in the full cell can be assigned to the cathode or the anode. Two equivalent cells were prepared as full cells, and then cycled galvanostatically (at C/10) until reaching the end of the first discharge, first charge or second discharge (discharged till 0.5). After cycling the cells were disassembled in the glovebox and reassembled as symmetric cells (cathode vs. cathode or anode vs. anode) with the used separators and electrolyte. (a-c) show the impedance response of the full and symmetric cells after the first discharge (a), first charge (b), and second discharge (c). Black curves in (a-d) show the full cell impedance. The insets show the high and middle frequency responses. (d) Curves represent the full cell response at high/middle frequency during first charge and second discharge. (e) Blue curves represent the cathode symmetric response of the full cells in (d). (f) Light blue curves represent the anode symmetric response of the full cells in (d). The data shows that at the mid-frequencies the cathode impedance is dominant.

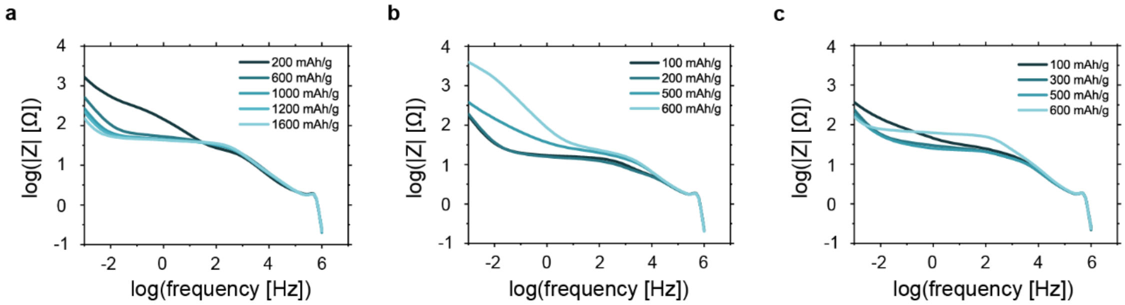

Figure S13: Bode plots of AC08-S 1-1 during (a) first discharge, (b) first charge and (c) second discharge.

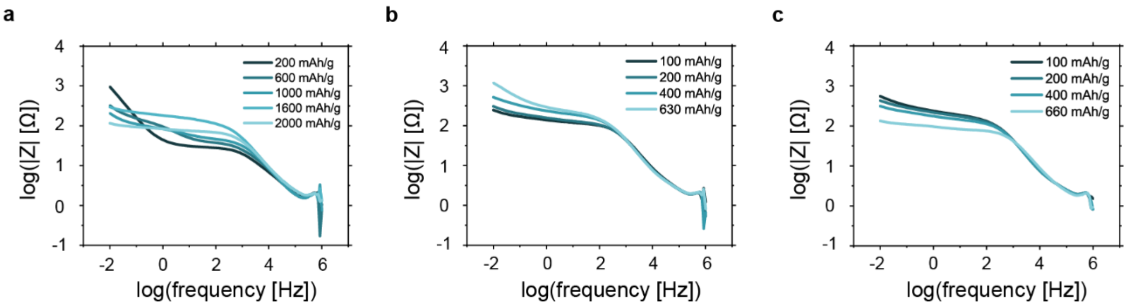

Figure S14: Bode plots of AC12-S 1-1 during (a) first discharge, (b) first charge and (c) second discharge.

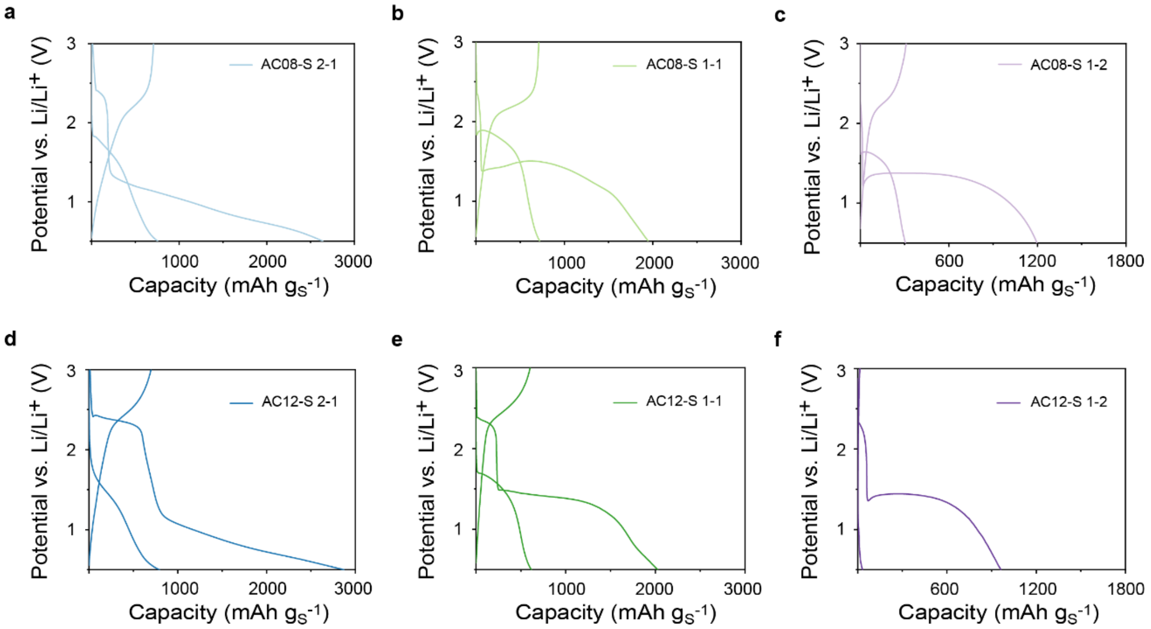

Figure S15: Galvanostatic first (dis)charge and second discharge curves of AC08-S and AC12-S electrodes with different S/C ratios. All measurements were conducted at room temperature. (a-c), AC08/S electrodes with C/S mass ratios of 2/1 (a), 1/1 (b), and 1/2 (c). (d-f), AC12/S electrodes with C/S mass ratios of 2/1 (d), 1/1 (e), and 1/2 (f). Note the different shapes of the charge/discharge curves, depending on the nanopore structure and sulfur mass loading.

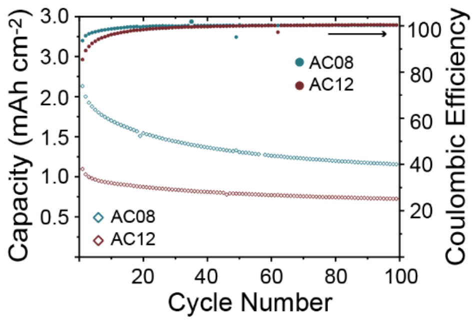

Figure S16: Long-term cycling data, based on capacity loadings. Galvanostatic discharge capacities (C/10) with different carbon indicated as color tones. Blue and red represent the pore size of 0.8 nm (AC08) and 1.2 nm (AC12), respectively at a C/S mass ratio of 1/1. Discharge capacities (left axis) and coulombic efficiencies (right axis). Note that the sulfur loading of the two electrodes was not identical, leading to the relatively large difference in initial areal capacities.

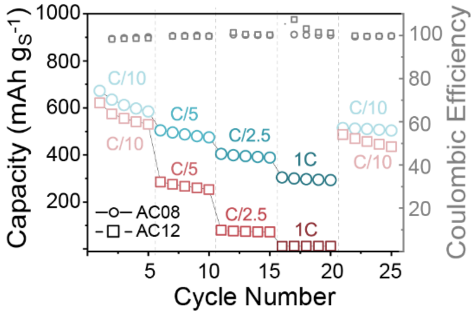

Figure S17: Rate capability data, based on gravimetric capacities. Galvanostatic (dis)charge curves with different (dis)charge rates indicated as color tones. Blue and red represent the pore size of 0.8 nm (AC08) and 1.2 nm (AC12), respectively at a C/S mass ratio of 1/1. Discharge capacities (left axis) and coulombic efficiencies (right axis) at different rates of C/10, C/5, C/2.5, 1C (with 5 cycles at a rate).

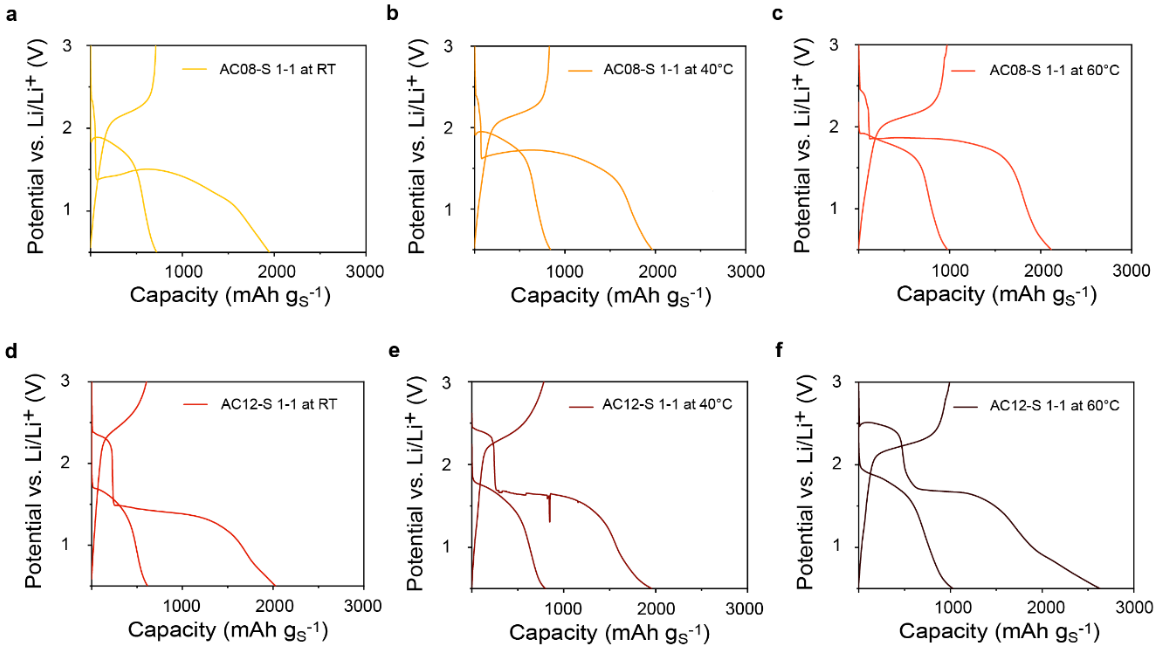

Figure S18: Galvanostatic cycling curves representing first (dis)charge and second discharge of AC08-S and AC12-S electrodes at different temperatures. All measurements were conducted with an AC/S mass ratio of 1/1. (a-c), AC08/S electrodes cycled at room temperature (a), 40°C (b), and 60°C (c). (d-f), AC12/S electrodes cycled at room temperature (d), 40°C (e), and 60°C (f). Note the different shapes of the charge/discharge curves and different capacities, depending on the nanopore structure (AC08 vs. AC12) and the measurement temperature.

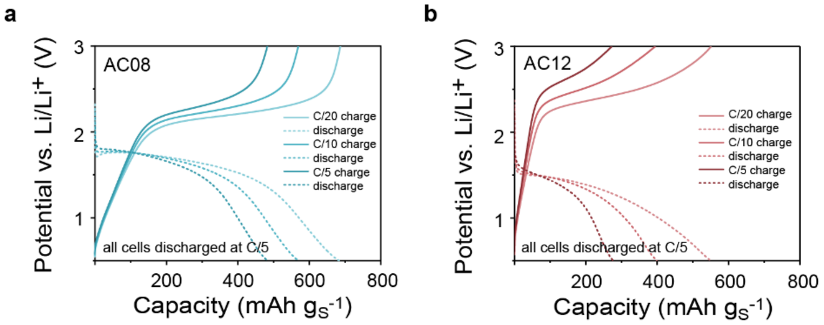

Figure S19: Rate performance data of AC08-S and AC12-S with a 1/1 C/S mass ratio. Galvanostatic charge curves with different rates followed by discharge curves indicated as color tones. (a) Blue and (b) red represent the pore size of 0.8 nm (AC08) and 1.2 nm (AC12), respectively. Specific capacities for constant discharge rates but varying charge rates for AC08 and AC12. All cells were discharged with C/5, the charge rates are varied from C/20 to C/5. By increasing the charge rate, the overpotential increased for both carbons.

2. Supporting Tables

Table S1: Scattering length densities (SLDs) of carbon, sulfur, lithium sulfide and possible CEI components.[1]

	Mass density (g cm^{-3})	Molar mass (g mol^{-1})	Molar volume (cm^3 mol^{-1})	SLD$_{SANS}$ (cm^{-2})
**Li_2S**	1.66	45.95	27.68	-0.21×10^{10}
**S**	2.07	32.06	15.49	1.07×10^{10}
**Carbon**	2.00	12	5.85	6.67×10^{10}
**FEC/DMC$_{deut}$ (1:4)**	1.11	97.76	87.91	4.78×10^{10}
**$LiPF_6$**	1.50	151.90	101.27	2.21×10^{10}
**1 M $LiPF_6$ in FEC/DMC$_{deut}$ (1:4)**	-	-	-	4.55×10^{10}
**Li_2O**	2.01	29.88	14.85	0.81×10^{10}
**LiF**	2.64	25.94	9.83	2.30×10^{10}
**Li_2CO_3**	2.11	73.89	35.02	3.48×10^{10}

3. Supporting Notes

Supporting Note 1

Discussion of EIS data during the first discharge:

The Nyquist plot of the first discharge displays an initially large arc at low-frequencies around 20 Hz and below. As this high-impedance feature occurs specifically during the beginning of the first discharge, we speculate that its origin is related to dissolved polysulfides and CEI formation[2,3]. As the CEI forms, the low-frequency arc becomes smaller, and the low-frequency trend changes its slope from a linear behavior (approximately a 45° slope) to a capacitive behavior (low-frequency trend close to a vertical line) at the end of the first discharge where the lithiation completes and Li_2S forms[4] (Figure S10c, FigureS11c).

4. Supporting References

(1) Neutron Scattering Lenghts and Cross Sections of the Elements and Their Isotopes. Neutron News, Vol.3, No.3, pp 29-37, 1992.
(2) Drvarič Talian, S.; Kapun, G.; Moškon, J.; Dominko, R.; Gaberšček, M. Transmission Line Model Impedance Analysis of Lithium Sulfur Batteries: Influence of Lithium Sulfide Deposit Formed During Discharge and Self-Discharge. *Journal of The Electrochemical Society* **2022**, *169* (1), 010529-010538. DOI: 10.1149/1945-7111/ac4a4e.
(3) Drvarič Talian, S.; Moškon, J.; Dominko, R.; Gaberšček, M. The Pitfalls and Opportunities of Impedance Spectroscopy of Lithium Sulfur Batteries. *Advanced Materials Interfaces* **2021**, *9* (8), 2101116-2101128. DOI: 10.1002/admi.202101116.
(4) Barchasz, C.; Leprêtre, J.-C.; Alloin, F.; Patoux, S. New Insights into the Limiting Parameters of the Li/S Rechargeable Cell. *Journal of Power Sources* **2012**, *199*, 322-330. DOI: 10.1016/j.jpowsour.2011.07.021.
